# A small molecular compound CC1007 induces cross-lineage differentiation by inhibiting HDAC7 expression and HDAC7/MEF2C interaction in BCR-ABL1^−^ pre-B-ALL

**DOI:** 10.1038/s41419-020-02949-1

**Published:** 2020-09-10

**Authors:** Zhihua Wang, Yang Zhang, Shicong Zhu, Hongling Peng, Yongheng Chen, Zhao Cheng, Sufang Liu, Yunya Luo, Ruijuan Li, Mingyang Deng, Yunxiao Xu, Guoyu Hu, Lin Chen, Guangsen Zhang

**Affiliations:** 1 Department of Hematology, The Second Xiangya Hospital, Central South University, Changsha, Hunan China; 2 Institute of Molecular Hematology, Central South University, Changsha, Hunan China; 3 Department of Oncology, The Second Xiangya Hospital, Central South University, Changsha, Hunan China; 4 Department of Geriatrics, The Second Xiangya Hospital, Central South University, Changsha, Hunan China; 5 Laboratory of Structural Biology, Key Laboratory of Cancer Proteomics of Chinese Ministry of Health, Xiangya Hospital & State Key Laboratory of Medical Genetics, Central South University, Changsha, Hunan China; 6 Department of Hematology, The Affiliated Zhuzhou Hospital of Xiangya Medical College, Central South University, Zhuzhou, Hunan China; 7 Molecular and Computational Biology Program, Department of Biological Sciences, University of Southern California, Los Angeles, CA 90089 USA

**Keywords:** Acute lymphocytic leukaemia, Translational research

## Abstract

Histone deacetylase 7 (HDAC7), a member of class IIa HDACs, has been described to be an important regulator for B cell development and has a potential role in B cell acute lymphoblastic leukemia (B-ALL). CC1007, a BML-210 analog, is designed to indirectly inhibit class IIa HDACs by binding to myocyte enhancer factor-2 (MEF2) and blocking the recruitment of class IIa HDACs to MEF2-targeted genes to enhance the expression of these targets. In this study, we investigated the anticancer effects of CC1007 in breakpoint cluster region-Abelson 1 fusion gene-negative (BCR-ABL1^−^) pre-B-ALL cell lines and primary patient-derived BCR-ABL1^−^ pre-B-ALL cells. CC1007 had obvious antileukemic activity toward pre-B-ALL cells in vitro and in vivo; it also significantly prolonged median survival time of pre-B-ALL-bearing mice. Interestingly, low dose of CC1007 could inhibit proliferation of BCR-ABL1^−^ pre-B-ALL cells in a time-dependent manner not accompanied by significant cell apoptosis, but along with cross-lineage differentiation toward monocytic lineage. From a mechanistic angle, we showed that HDAC7 was overexpressed in BCR-ABL1^−^ pre-B-ALL cells compared to normal bone marrow samples, and CC1007 could reduce the binding of HDAC7 at the promoters of monocyte–macrophage-specific genes via inhibition of HDAC7 expression and HDAC7:MEF2C interaction. These data indicated that CC1007 may be a promising agent for the treatment of BCR-ABL1^−^ pre-B-ALL.

## Introduction

B lymphopoiesis is a complicated process that takes place in a step-by-step manner and involves several cellular transitions, including cell commitment and differentiation. Each cellular transition is strictly regulated at the transcriptional level by the action of linage-specific transcription factors (TFs), such as PU.1, Ikaros, myocyte enhancer factor-2 (MEF2C), E2A, and PAX5^1–6^. Deregulation of these particularly transcriptional programs may lead to blockage of B cell differentiation and uncontrolled cell proliferation, thus conducing to the development of hematological malignancies, such as leukemia and lymphoma. Aberrant expression, mutation, rearrangement, and translocation of these lineage-specific TFs implicated in B lymphocyte development have been associated with hematopoietic tumorigenesis^7–10^.

In addition to genetic alterations, the development and progression of cancer are related to changes in epigenetic mechanisms^11–13^. The two main epigenetic mechanisms are the DNA methylation and histone modifications^14,15^. Among the mechanisms of histone modifications, histone acetylation is the most studied and is regulated by histone acetyltransferase and histone deacetylase (HDAC). Mutation and/or aberrant expression of HDACs have often been observed in many cancers and a corresponding abnormal acetylation of histones, resulting in altered expression and activity of numerous proteins implicated in carcinogenesis and making them important therapeutic targets^16–21^. Among these HDACs, HDAC7 appears to be a lymphoid-specific transcriptional suppressor^22–27^. In addition to its important role in T lymphocyte biology, HDAC7 could be an important regulator of B cell development and is critical in maintaining the genetic properties of B lymphocytes^16^. In addition, HDAC7 has been identified as a target gene in hematopoietic tumors in a PiggyBac transposon mutagenesis screening in mice^28^. HDAC7 expression level is higher in childhood ALL when compared to normal bone marrow (BM) samples and higher expression of HDAC7 is associated with poor prognosis in B-lineage CD10^+^ patients^29^. Here, our data showed that HDAC7 is overexpressed in both breakpoint cluster region-Abelson 1 fusion gene-negative (BCR-ABL1^−^) and BCR-ABL1^+^ B cell acute lymphoblastic leukemia (B-ALL) cells when compared to normal BM samples. Based on these findings, we postulated that HDAC7 might be an important therapeutic target for pre-B-ALL.

**Fig. 1:**
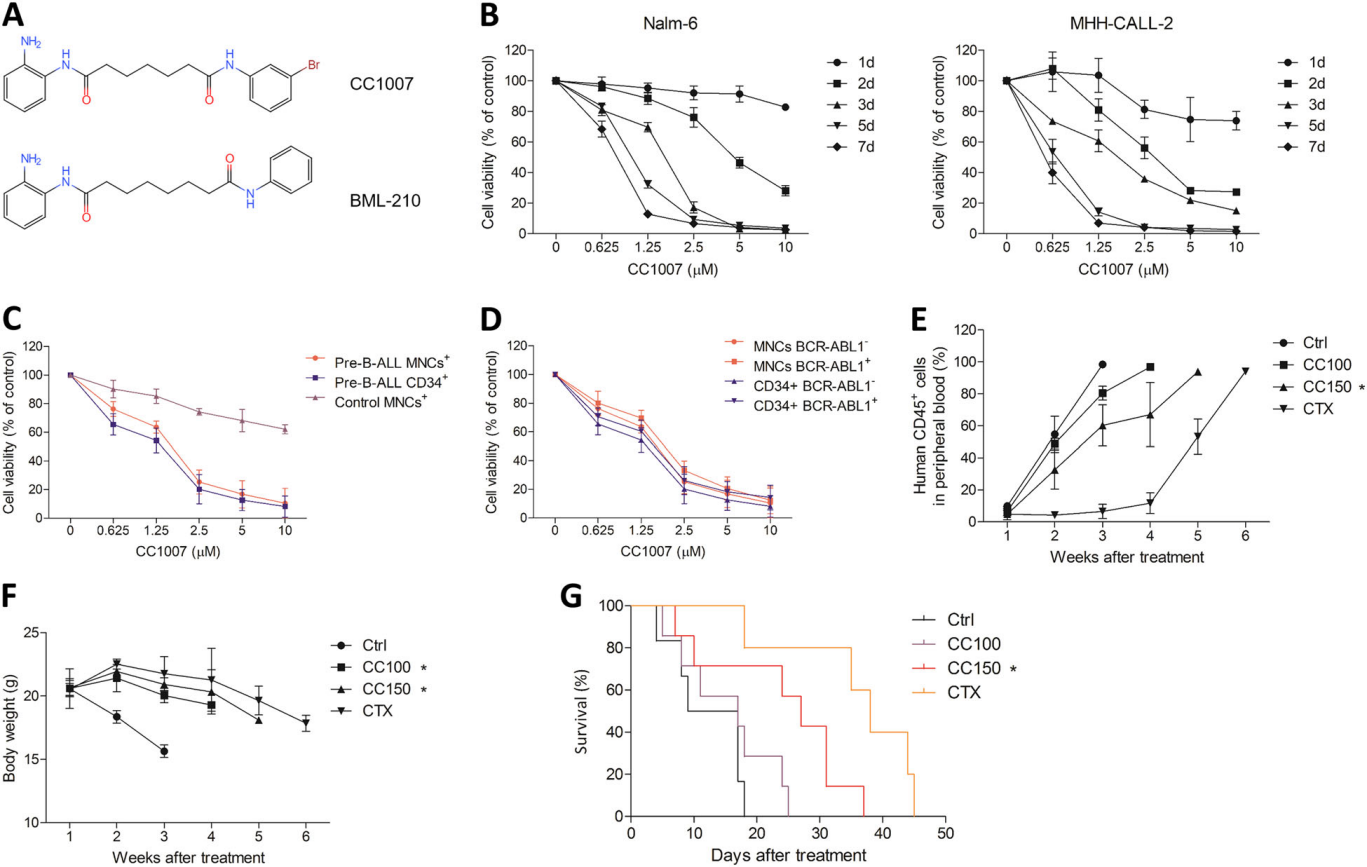
Antiproliferative effect of CC1007 in vitro and in vivo. **a** Chemical structure of CC1007 and BML-210. **b** Effects of CC1007 on BCR-ABL1^−^ pre-B-ALL cell line Nalm-6 and MHH-CALL-2 cell growth (MTT assay). The cell growth curve represents the effect of CC1007 at different concentrations for 7 days. The values represent the mean ± SEM of triplicate cultures. **c** Effects of CC1007 on fresh CD34^+^ cell growth from BCR-ABL1^−^ pre-B-ALL patients (*n* = 15), fresh mononuclear cell (MNC) growth from BCR-ABL1^−^ pre-B-ALL patients (*n* = 20), and fresh MNC growth from normal controls (*n* = 10). Cells were treated with CC1007 for 72 h, and then cell viability was evaluated by the MTT assay. **d** Effect of CC1007 on BCR-ABL1^+^ primary CD34^+^ cell (*n* = 8) or MNC (*n* = 8) growth and BCR-ABL1^−^ primary CD34^+^ cell (*n* = 15) or MNC (*n* = 20) growth from pre-B-ALL patients. Cells were treated with CC1007 for 72 h. The growth curve represents the effect of CC1007 at different concentrations, which are shown as the mean ± SEM. **e** Examination was performed on human white blood cell (WBC) antigen (CD45) levels to evaluate the effect of CC1007 on the proliferation of human primary BCR-ABL1^−^ pre-B-ALL cells in a xenograft model. The results represent the mean ± SEM. **f** Changes of body weight of mice treated with CC1007 and CTX. The results represent the mean ± SEM. **g** A Kaplan–Meier survival plot for BCR-ABL1^−^ pre-B-ALL-bearing NOD/SCID mice is shown. The survival curves differed significantly between the CC1007 (150 mg/kg)-treated group and control group (*P* < 0.05; log-rank test). Ctrl control, CC100 CC1007 100 mg/kg, CC150 CC1007 150 mg/kg, CTX cyclophosphamide. Statistical significance was determined by two-tailed Student’s *t* tests. Asterisk denotes significant (*P* < 0.05) differences relative to the control.

CC1007, a BML-210 analog (Fig. 1a), is designed to indirectly inhibit class IIa HDACs by binding to MEF2 and blocking the recruitment of class IIa HDACs to MEF2-targeted genes to enhance the expression of these targets^30^. HDAC7, a member of class IIa HDACs, could interact with MEF2C in pre-B cells and B cell malignancies^22,31^. We therefore sought to investigate whether CC1007 has antileukemic effects against BCR-ABL1^−^ pre-B-ALL. We observed that CC1007 can inhibit BCR-ABL1^−^ pre-B-ALL cells growth, induce cell cycle arrest, and cross-lineage differentiation. In addition, we found that these effects of CC1007 on BCR-ABL1^−^ pre-B-ALL cells were partially due to suppression of HDAC7 and interference in the interaction of HDAC7/MEF2C. Furthermore, we showed that CC1007 could significantly prolong median survival time of primary BCR-ABL1^−^ pre-B-ALL-bearing mice, suggesting that CC1007 may represent an alternative molecular targeting strategy for BCR-ABL1^−^ pre-B-ALL treatment.

## Results

### CC1007 inhibits proliferation in pre-B-ALL cells in vitro and in vivo

The antileukemic effects of CC1007 in pre-B-ALL cells were evaluated by the MTT [3-(4,5-dimethylthiazol-2-yl)-2,5-diphenyltetrazolium bromide] assay. CC1007 inhibited the growth of BCR-ABL1^−^ pre-B-ALL cell line Nalm-6 and MHH-CALL-2 (Fig. 1b). Moreover, we confirmed that CC1007 exhibited an overt growth-inhibiting effect on primary CD34^+^ BCR-ABL1^−^ pre-B-ALL cells, corresponding to BCR-ABL1^−^ pre-B-ALL mononuclear cells (MNCs) (Fig. 1c and Table 1). It is noteworthy that CC1007 had a minor cytotoxic effect on normal MNCs (Fig. 1c). In addition, we observed that BCR-ABL1^−^ primary pre-B-ALL cells were more sensitive to CC1007 than BCR-ABL1^+^ cells (Fig. 1d and Table 2). Moreover, we established pre-B-ALL xenograft nonobese diabetic/severe combined immunodeficiency (NOD/SCID) model using primary MNCs from one BCR-ABL1^−^ pre-B-ALL patient who overexpressed HDAC7 (Supplementary Fig. 1) for testing the antitumor activity of CC1007 in vivo. The results showed CC1007 inhibited the development of leukemic cells in vivo and reduced the tumor burden of xenograft mice without significant weight loss, when compared to untreated group (Fig. 1e–f and Supplementary Fig. 2). Kaplan–Meier analysis indicated that CC1007 could prolong median survival time of leukemia-bearing mice (12.2 ± 5.9 days for untreated mice versus 15.4 ± 7.7 and 23.9 ± 11.3 days for mice treated with CC1007 at 100 and 150 mg/kg per day, respectively) (Fig. 1g). These results confirmed that CC1007 possessed antileukemia potential for human pre-B-ALL in vitro and in vivo.

**Table 1:** IC_50_ values for CC1007 on human primary BCR-ABL1^−^ pre-B-ALL cells and normal MNCs.

	IC_50_ (μM)
Pre-B-ALL MNCs (20 samples)	1.546 ± 0.812*
Pre-B-ALL CD34^+^ (15 samples)	1.129 ± 0.632
Control MNCs (10 samples)	19.923 ± 3.531

IC_50_ (μM) ± SEM: The drug concentration that inhibited cell survival by 50% (mean ± SEM).

*Significant (*P* < 0.05) differences between pre-B-ALL MNCs and pre-B-ALL CD34^+^.

**Table 2:** IC_50_ values for CC1007 on human primary pre-B-ALL cells.

	IC_50_ (μM)
MNCs BCR-ABL1^−^ (20 samples)	1.546 ± 0.812*
MNCs BCR-ABL1^+^ (8 samples)	1.890 ± 0.769
CD34^+^ BCR-ABL1^−^ (15 samples)	1.129 ± 0.632^#^
CD34^+^ BCR-ABL1^+^ (8 samples)	1.421 ± 0.629

IC50 (μM) ± SEM: The drug concentration that inhibited cell survival by 50% (mean ± SEM).

*Significant (*P* < 0.05) differences between MNCs BCR-ABL1^−^ and MNCs BCR-ABL1^+^.

^#^Significant (*P* < 0.05) differences between CD34^+^ BCR-ABL1^−^ and CD34^+^ BCR-ABL1^+^.

### Low dose of CC1007 hardly induces pre-B-ALL cells apoptosis

**Fig. 2:**
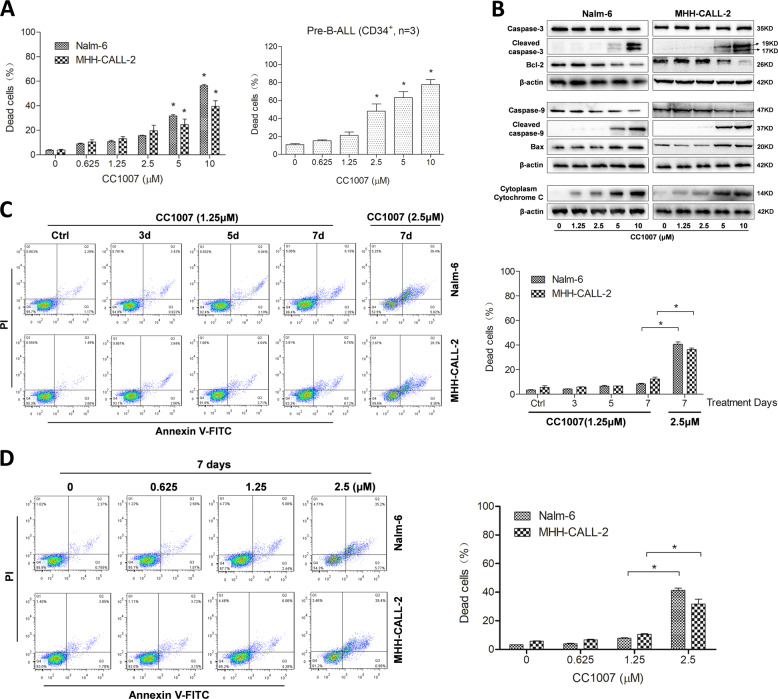
Low dose of CC1007 hardly induces pre-B-ALL cells apoptosis. **a** BCR-ABL1^−^ pre-B-ALL cell lines Nalm-6 and MHH-CALL-2 and primary CD34^+^ cells from BCR-ABL1^−^ pre-B-ALL patients were treated with increasing concentrations of CC1007 for 48 h, which was followed by an analysis of apoptosis by staining with PI and Annexin V FITC. Annexin V-positive cells were measured by flow cytometry. Columns represent the average percent of Annexin V-positive cells from three independent experiments, which are shown as the mean ± SEM. Asterisks indicate statistically significant (*P* < 0.05) differences compared with controls by one-way ANOVA. **b** Effects of CC1007 on caspase-3, caspase-9, Bcl-2, Bax, and cytoplasm cytochrome *C* expression (western blot results). β-Actin is used as a loading control. **c**, **d** Nalm-6 and MHH-CALL-2 cells were treated with low dose of CC1007 (0.625 and 1.25 μM) or 2.5 μM of CC1007 for 7 days. Cell apoptosis was measured by flow cytometry. Columns represent the average percent of Annexin V-positive cells from three independent experiments, which are shown as the mean ± SEM. Representative images are shown in the left panel.

To confirm whether the antitumor activity of CC1007 is associated with apoptosis, we evaluated cell apoptosis using Annexin V staining. Our results showed high dose of CC1007-induced apoptosis in Nalm-6 and MHH-CALL-2 cells and primary CD34^+^ BCR-ABL1^−^ pre-B-ALL cells (Fig. 2a and Supplementary Fig. 3). In addition, we assessed the expression and activation of apoptosis-related proteins. As shown in Fig. 2b, CC1007 upregulated the expression of Bax and downregulated Bcl-2 protein levels, which was accompanied by the cleavage of caspase-3 and caspase-9, and increased the release of cytochrome *C*. Surprisingly, low dose of CC1007 (≤1.25 μM) hardly induced BCR-ABL1^−^ pre-B-ALL cells apoptosis and did not influence the expression of apoptosis-regulating molecules even after 7 days of treatment (Fig. 2a–d).

### CC1007 arrests cell cycle at G0/G1 phase in pre-B-ALL cell lines

**Fig. 3:**
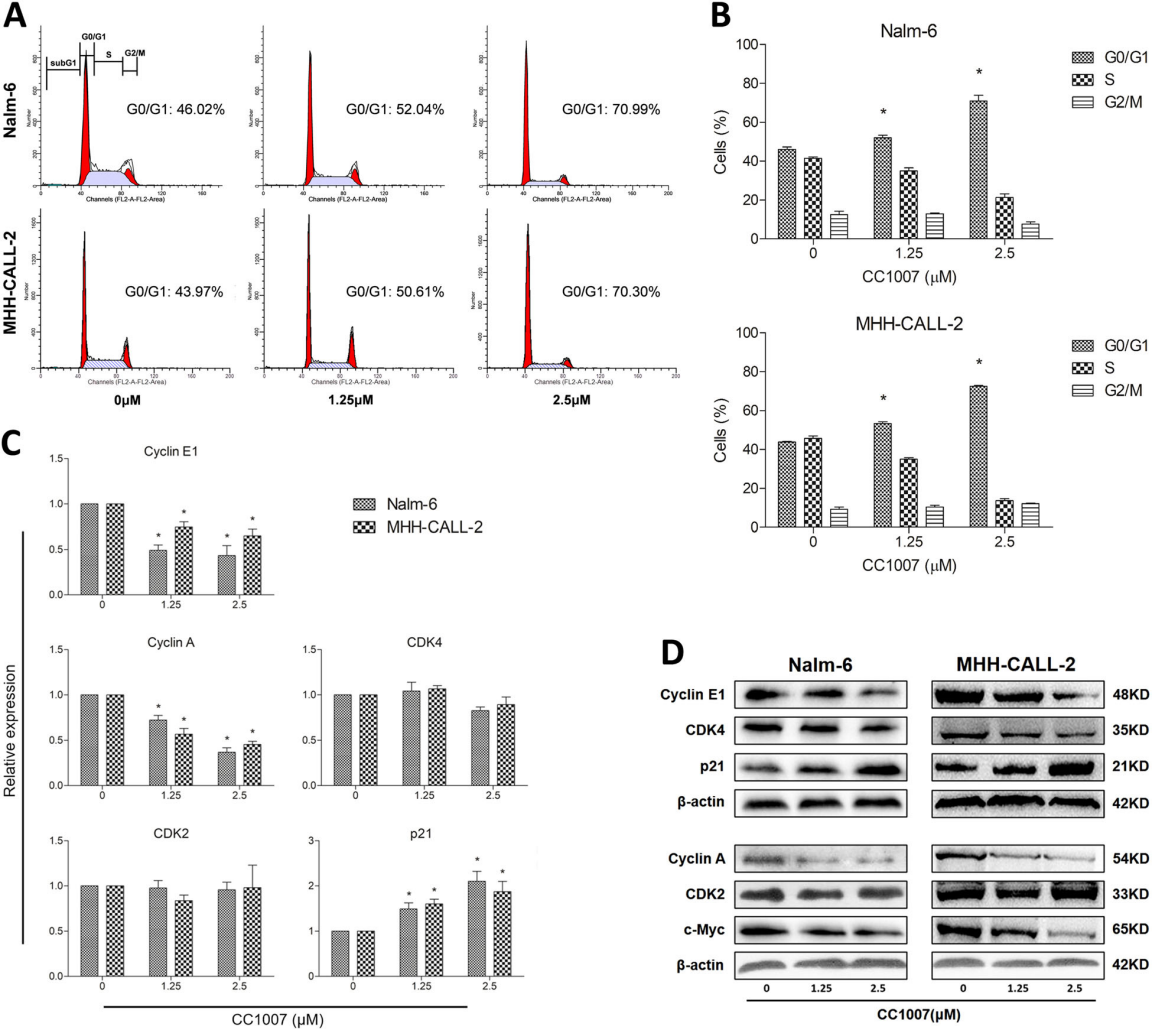
Effects of CC1007 on cell cycle progression and related regulators. **a** A representative cell cycle analysis performed by flow cytometry of Nalm-6 and MHH-CALL-2 cells treated with different doses of CC1007 for 48 h is shown. **b** The percentage of cells in the G0/G1 phase of the cell cycle after CC1007 treatment for 48 h is shown. The data represent the mean ± SEM of three different experiments. **c**, **d** mRNA and proteins levels of CDK4, CDK2, cyclin A, cyclin E1, c-Myc, and p21 were analyzed by RT-qPCR and western blotting after 48 h of treatment, respectively. β-Actin was used as a loading control. The results are representative of three independent experiments. Asterisks denote statistically significant (*P* < 0.05) differences compared with controls by one-way ANOVA.

Next, we examined the effects of CC1007 on cell cycle progression in Nalm-6 and MHH-CALL-2 cells. The results indicated that CC1007 might significantly arrest the cell cycle at the G0/G1 phase (Fig. 3a, b). We evaluated the changes in G0/G1- to S-phase transition-related regulatory proteins to uncover the molecular events of cell cycle arrest. Cyclin-dependent kinase 4 (CDK4) is necessary for transition through the G1 phase, whereas CDK2, cyclin E, and cyclin A are necessary for completion of the G1 phase and initiation of the S phase^32^. As indicated in Fig. 3c, d, CC1007 downregulated cyclin E and cyclin A, but it had little effect on CDK4 and CDK2. Meanwhile, the expression of CDK4 inhibitor p21 increased under CC1007 treatment. CC1007 also depressed the expression of c-Myc, which is responsible for cell cycle arrest in the G1 phase. These results documented that CC1007 could induce G0/G1 arrest in pre-B-ALL cells.

### CC1007 induces BCR-ABL1^−^ pre-B-ALL cells cross-lineage differentiation

**Fig. 4:**
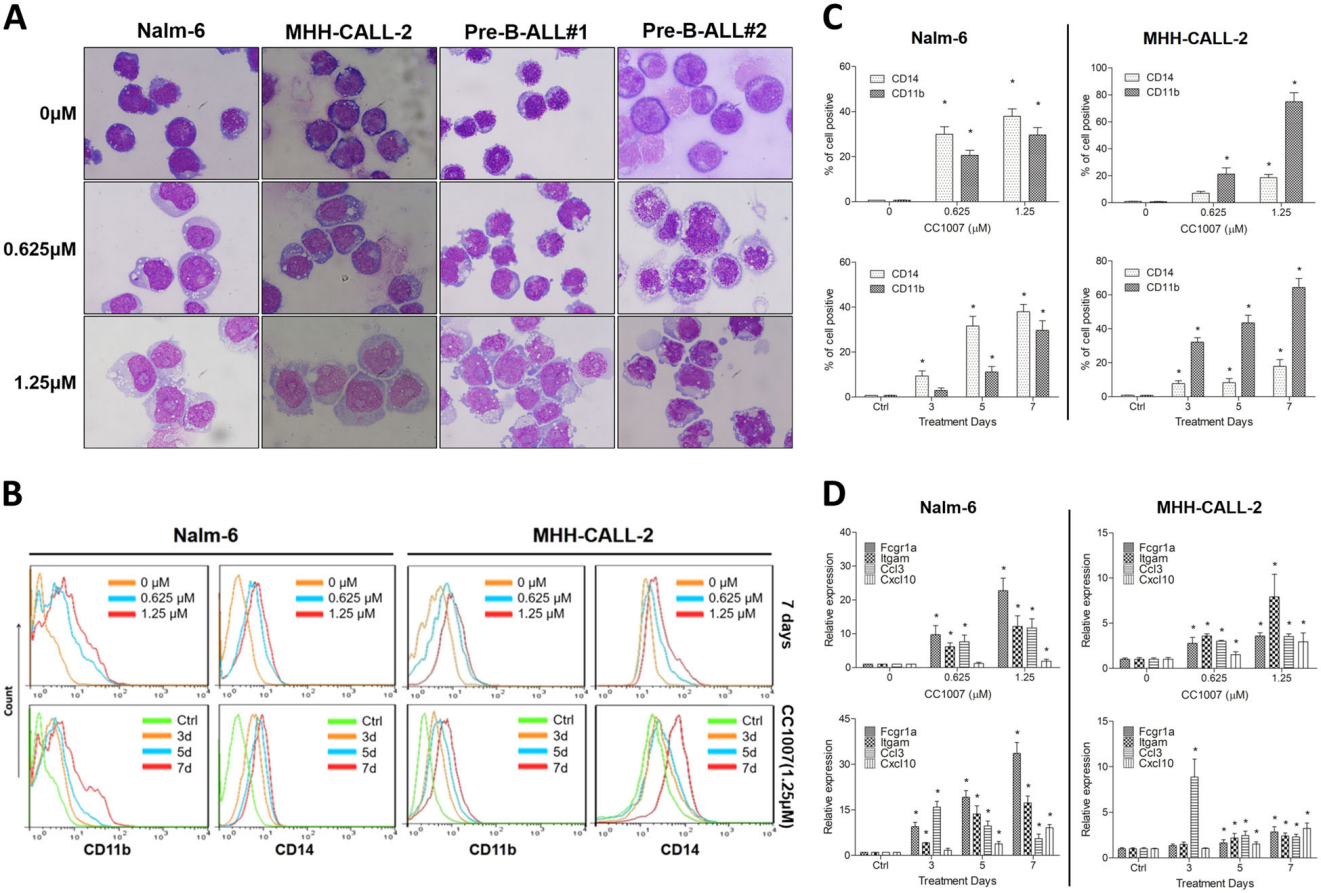
CC1007 induces BCR-ABL1^−^pre-B-ALL cells cross-lineage differentiation. **a** Representative Wright–Giemsa staining for morphological examination is shown. Nalm-6 and MHH-CALL-2 cells and primary CD34^+^ BCR-ABL1^−^ pre-B-ALL cells were treated with low dose of CC1007 (0.625 and 1.25 μM) for 7 days. Original magnification was ×4000 (objective lens 100) under a light microscope (Olympus BX-50 microscope), and images were captured using the DP Controller software (Olympus) at room temperature. **b**, **c** Nalm-6 and MHH-CALL-2 cells were treated with low dose of CC1007 (0.625 and 1.25 μM) for 7 days. The expression of CD11b and CD14 in Nalm-6 and MHH-CALL-2 cells is shown. The percentage of cells expressing CD11b and CD14 was detected by flow cytometry analyses. The data represent the mean ± SEM of three different experiments. **d** Cells were treated with low dose of CC1007 (0.625 and 1.25 μM) for 7 days. RT-qPCR experiments for gene expression changes of *Fcgr1*, *Itgam*, *Ccl3*, and *Cxcl10* induced by CC1007 in Nalm-6 and MHH-CALL-2 cells. The data represent the mean ± SEM of three different experiments. Asterisks denote statistically significant (*P* < 0.05) differences compared with controls by one-way ANOVA.

We observed the morphologic alterations of pre-B-ALL cell lines Nalm-6 and MHH-CALL-2 and primary CD34^+^ BCR-ABL1^−^ pre-B-ALL cells undergoing low dose of CC1007 treatment. We found that CC1007 could induce BCR-ABL1^−^ pre-B-ALL cells differentiation toward monocytes. These cells displayed monocyte-like morphological characteristics, including increased cell volume and a lower nucleo/cytoplasmic ratio than pre-B-ALL cells, grayish cytoplasm, and irregular nuclei with a folded appearance (Fig. 4a). We checked surface markers of myeloid differentiation (CD11b) and monocytic maturation (CD14) to further objectively confirm this phenomenon. As shown in Fig. 4b, c, the percentage of CD14^−^ and CD11b^+^ cells in BCR-ABL1^−^ pre-B-ALL cells exposed to CC1007 increased significantly. We also showed that the expression of Fcgr1a, Itgam, Ccl3, and Cxcl10, which are involved in the regulation of phagocytosis and immune modulation of monocytes, displayed inductive overexpression in a dose- and time-dependent manner after low dose of CC1007 treatment (Fig. 4d), suggesting that CC1007 could induce cross-lineage differentiation in BCR-ABL1^−^ pre-B-ALL cells.

### HDAC7 is involved in CC1007-induced BCR-ABL1^−^ pre-B-ALL cells cross-lineage differentiation

**Fig. 5:**
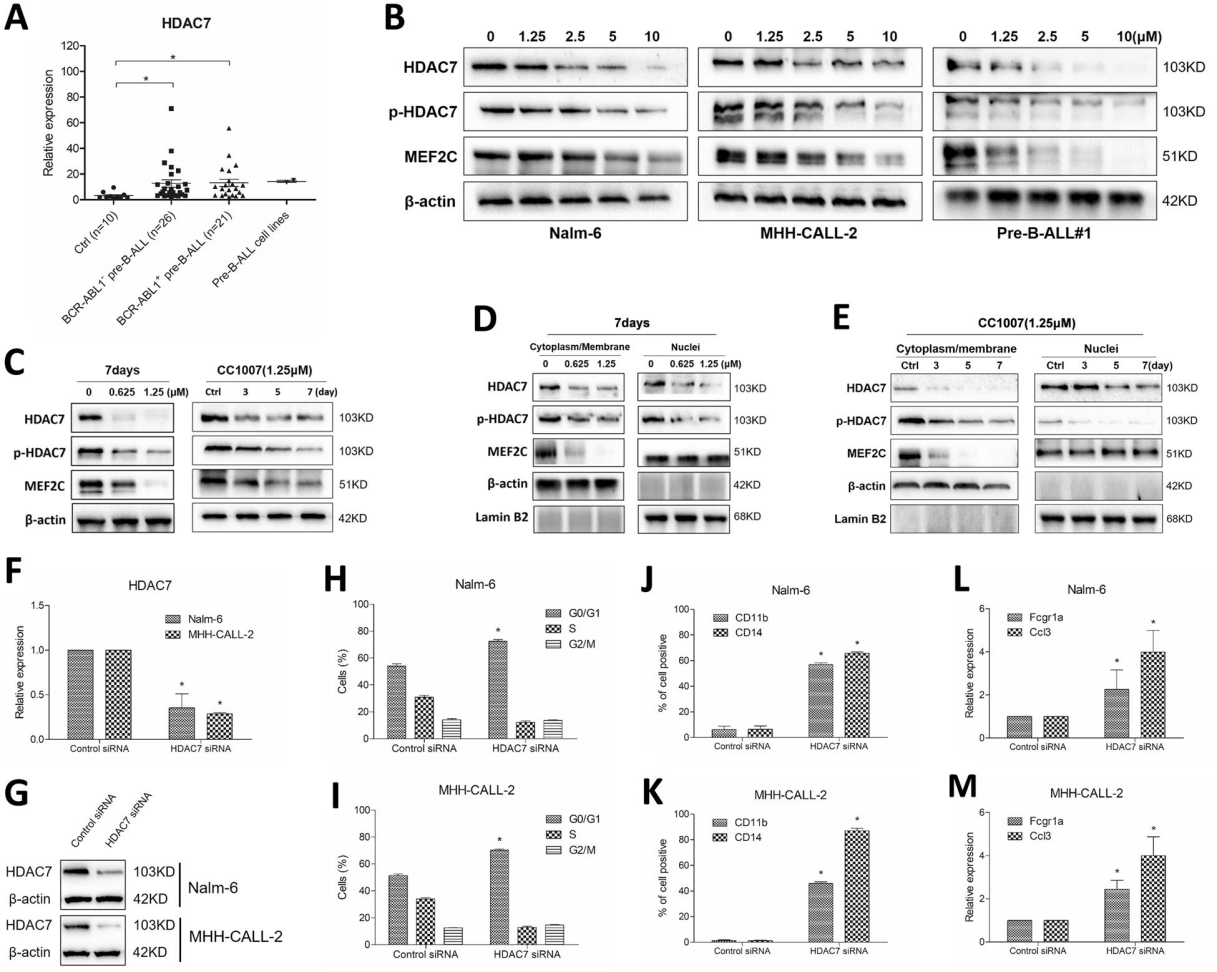
HDAC7 is involved in CC1007-induced BCR-ABL1^−^ pre-B-ALL cells cross-lineage differentiation. **a** Expression of HDAC7 in Nalm-6, MHH-CALL-2, BCR-ABL1^−^, and BCR-ABL1^+^ pre-B-ALL MNCs (*n* = 47) and normal MNCs (*n* = 10). Asterisks denote statistically significant (*P* < 0.05) differences compared with controls by two-tailed Student’s *t* tests. **b** CC1007 inhibits HDAC7 and MEF2C expression. Nalm-6, MHH-CALL-2, and primary BCR-ABL1^−^ pre-B-ALL cells were treated with increasing doses of CC1007 for 48 h. Whole-cell extracts were analyzed by western blotting for MEF2C and HDAC7 protein using β-actin as a loading control. **c** Nalm-6 cells were treated with low dose of CC1007 (0.625 and 1.25 μM) for 7 days. Whole-cell extracts were analyzed by western blotting for MEF2C and HDAC7 protein using β-actin as a loading control. **d**, **e** The effect of CC1007 on the subcellular distribution of MEF2C and HDAC7. Nalm-6 cells were treated with low dose of CC1007 (0.625 and 1.25 μM) for 7 days. The cytoplasmic/membrane and nuclear fractions of the cells were analyzed by western blotting for MEF2C, HDAC7, and p-HDAC7 proteins with β-actin and lamin B2 as cytoplasmic and nuclear loading controls, respectively. **f**, **g** Nalm-6 and MHH-CALL-2 cells were transfected with control siRNA or siRNAs targeting HDAC7, mRNA, and protein levels were examined by RT-qPCR and Western blot experiments 72 hours after siRNA transfection, respectively. **h**, **i** The percentages of cells in G0/G1 phases of the cell cycle are shown 72 h after transfection. **j**, **k** CD11b^+^/CD14^+^ cells were measured by flow cytometry 72 h after transfection. **l**, **m** *Fcgr1a* and *Ccl3* expression was assessed by RT-qPCR 72 h after transfection. The data represent the mean ± SEM of three different experiments. Statistical significance was determined by two-tailed Student’s *t* tests. Asterisk denotes significant (*P* < 0.05) differences relative to the control.

Several reports have described that HDAC7, as a member of class IIa HDACs, could be an important regulator for B cell development and a potential role in B-ALL^22,29,31,33^. Furthermore, HDAC7 specifically interacts with MEF2C, and its downregulation leads to differentiation toward macrophages in pre-B cells^22^. Accordingly, we assessed the mRNA level of HDAC7 on BCR-ABL1^−^ pre-B-ALL cell lines, primary pre-B-ALL MNCs (patient samples are listed in Supplementary Table 3), and healthy individuals. The results showed that HDAC7 was overexpressed in both BCR-ABL1^−^ and BCR-ABL1^+^ primary pre-B-ALL cells, when compared to healthy individuals (Fig. 5a). Moreover, when BCR-ABL1^−^ pre-B-ALL cells were exposed to CC1007, HDAC7 and MEF2C protein levels were downregulated, even when exposed to low dose of CC1007 (Fig. 5b, c). The above-mentioned results were also proven by subcellular localization assay for HDAC7 and MEF2C distributions, where both nuclear and cytoplasmic HDAC7 protein were significantly inhibited when BCR-ABL1^−^ pre-B-ALL cells were treated with low dose of CC1007 (Fig. 5d, e), but nuclear MEF2C protein level was unchanged, suggesting that the distribution of HDAC7 may be an important determinant for the differentiation of Nalm-6 cells along the monocytic lineage. HDAC7 has been reported to dissociate from MEF2C, bind to its partner proteins, such as CaMK1, and translocate from the nucleus to the cytoplasm upon phosphorylation^34^. Our study illustrated that CC1007 could decrease total p-HDAC7 levels (Fig. 5b, c) and further experiments showed p-HDAC7 levels both in the cytoplasm and in the nuclei were reduced simultaneously after CC1007 treatment (Fig. 5d, e), implying that decrease of p-HDAC7 may be due to decreased total HDAC7 levels and whether HDAC7 phosphorylation was involved in the differentiation induced by CC1007 needs to be further studied. To determine the role of HDAC7 in CC1007-induced cell cycle arrest and differentiation in BCR-ABL1^−^ pre-B-ALL cells, we performed a loss-of-function experimental approach. We knocked down HDAC7 by small-interfering RNA (siRNA) in both Nalm-6 and MHH-CALL-2 cells. The efficacy of siRNA to inhibit HDAC7 is shown in Fig. 5f, g. After transfection, G0/G1-phase arrest was increased, as observed previously (Fig. 5h, i). CD11b and CD14 expression in Nalm-6 and MHH-CALL-2 cells (Fig. 5j, k) were increased in HDAC7 siRNA groups. Strikingly, we observed that the reduction in HDAC7 mRNA levels resulted in the derepression of key monocyte–macrophage genes such as *Fcgr1a* and *Ccl3* (Fig. 5l, m). These data demonstrate that HDAC7 plays an important role in BCR-ABL1^−^ pre-B-ALL cells cross-lineage differentiation induced by CC1007.

### CC1007 inhibits HDAC7:MEF2C interaction in the nucleus

**Fig. 6:**
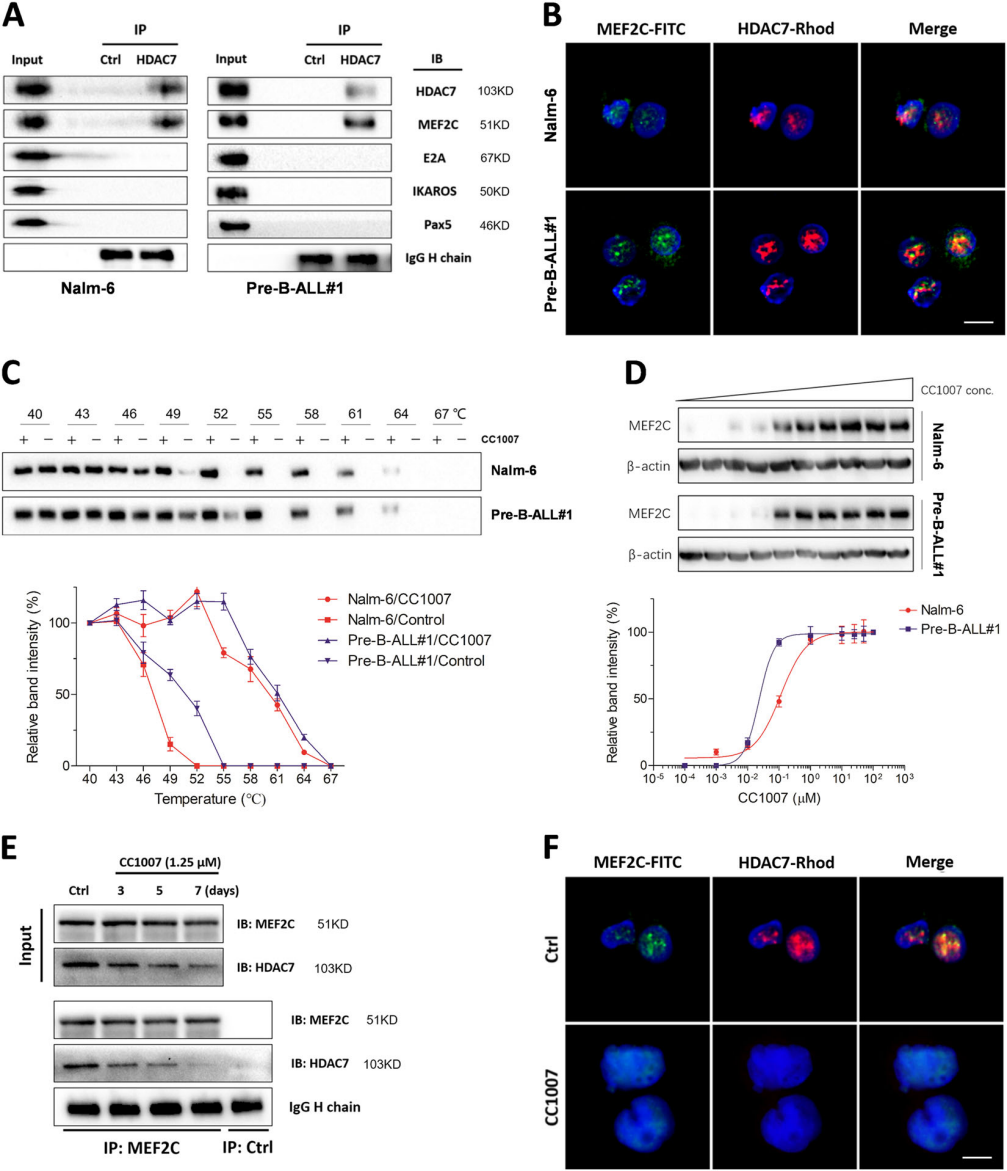
CC1007 blocks HDAC7 interaction with MEF2C. **a** Co-immunoprecipitation experiments showing the specific binding of HDAC7 with MEF2C in BCR-ABL1^−^ pre-B-ALL cells. HDAC7 does not bind with *IKAROS*, *Pax5*, or *E2A*. **b** BCR-ABL1^−^ pre-B-ALL cells were immunostained with anti-MEF2C antibody followed and anti-HDAC7 antibody, followed by corresponding FITC-conjugated anti-IgG secondary antibody and rhodamine-conjugated anti-IgG secondary antibody to show MEF2C protein and HDAC7 protein, respectively. Simultaneously, cells were stained with PI to display nuclei. The fluorescent images were visualized with a confocal microscope (Leica TCS SP5 II microscope) with (Leica Application Suite Advanced Fluorescence) acquisition software (Leica) at room temperature. (original magnification ×4000). Scale bars, 10 μm. **c**, **d** CC1007 interacts with MEF2C in BCR-ABL1^−^ pre-B-ALL cells. CETSA was performed on Nalm-6 cells and primary BCR-ABL1^−^ pre-B-ALL cells as described in the “Materials and methods” section. The stabilizing effects of CC1007 on MEF2C at different temperatures and different doses were evaluated by western blot analysis. The intensity of the MEF2C bands was quantified using the Image Lab™ software (Bio-Rad). For the CETSA curves, the band intensities were related to the intensities of the lowest temperatures for the drug-exposed samples and control samples. For the ITDRF_CETSA_ experiments, the band intensities were related to the control samples. Representative images are shown in the upper panel. **e** Nalm-6 cells were treated with 1.25 μM CC1007 for 7 days, co-immunoprecipitation experiments were performed on the third, fifth, and seventh day to evaluate the effect of CC1007 on the interaction between MEF2C and HDAC7, and **f** cells were immunostained after treatment on the seventh day as described in **b**. Original magnification ×4000. Scale bars, 10 μm. IP immunoprecipitation, IB immunoblotting, Rhod rhodamine.

We performed co-immunoprecipitation experiments to test the potential interaction genes of HDAC7, including *Pax5*, *IKAROS*, *E2A*, and *MEF2C*, and address whether HDAC7 interacts with particularly sequence-specific transcriptional factors in BCR-ABL1^−^ pre-B-ALL cells. The results indicated that HDAC7 specifically binds to MEF2C, but not to other B cell transcription-related genes (Fig. 6a). The same results were also observed with primary BCR-ABL1^−^ pre-B-ALL cells (Fig. 6a). The interaction of HDAC7 and MEF2C was confirmed by capturing fluorescence images using a confocal laser scanning microscope, where HDAC7 and MEF2C were mainly located in the nuclei and formed the HDAC7:MEF2C complex (Fig. 6b). Cellular thermal shift assay (CETSA) was employed to monitor the cellular target engagement of CC1007 and verify whether CC1007 could directly interact with MEF2C in BCR-ABL1^−^ pre-B-ALL cells. Compared with dimethyl sulfoxide (DMSO), CC1007 markedly increased MEF2C accumulation in the soluble fraction at the examined temperatures (Fig. 6c). We also tested the dose–response of CC1007 on the stability of MEF2C to heating. The dose–response study was conducted at 52 °C, a temperature at which a major portion of MEF2C was denatured and precipitated unless it was thermally stabilized by ligands. Increased stability of MEF2C was observed with increasing concentrations of CC1007 at 52 °C in an dose-dependent manner, suggesting that these effects are indeed thermally induced (Fig. 6d). These data indicate that CC1007 directly interacts with intracellular MEF2C. When BCR-ABL1^−^ pre-B-ALL cells were exposed to low dose of CC1007 (1.25 μM) for 7 days and co-immunoprecipitation was done in parallel on the third, fifth, and seventh day, the binding of HDAC7 and MEF2C became gradually weak and followed incubation-time prolongation (Fig. 6e), which was consistent with the immunofluorescence localization results where the color yellow indicated that the HDAC7:MEF2C complex was observed in the nuclei in the absence of CC1007 (Fig. 6f). After exposure to CC1007 (1.25 μM) for 7 days, precipitation of HDAC7: MEF2C was not detected (Fig. 6f). We speculated that diminution of the HDAC7:MEF2C complex may result from HDAC7 protein inhibition and/or CC1007-driven dissociation of the HDAC7:MEF2C complex. These results suggest that CC1007-induced trans-differentiation on Nalm-6 cells is associated with inhibition of HDAC7 and HDAC7:MEF2C interaction.

### CC1007 inhibits HDAC7:MEF2C interaction on the promotor of monocyte–macrophage genes

**Fig. 7:**
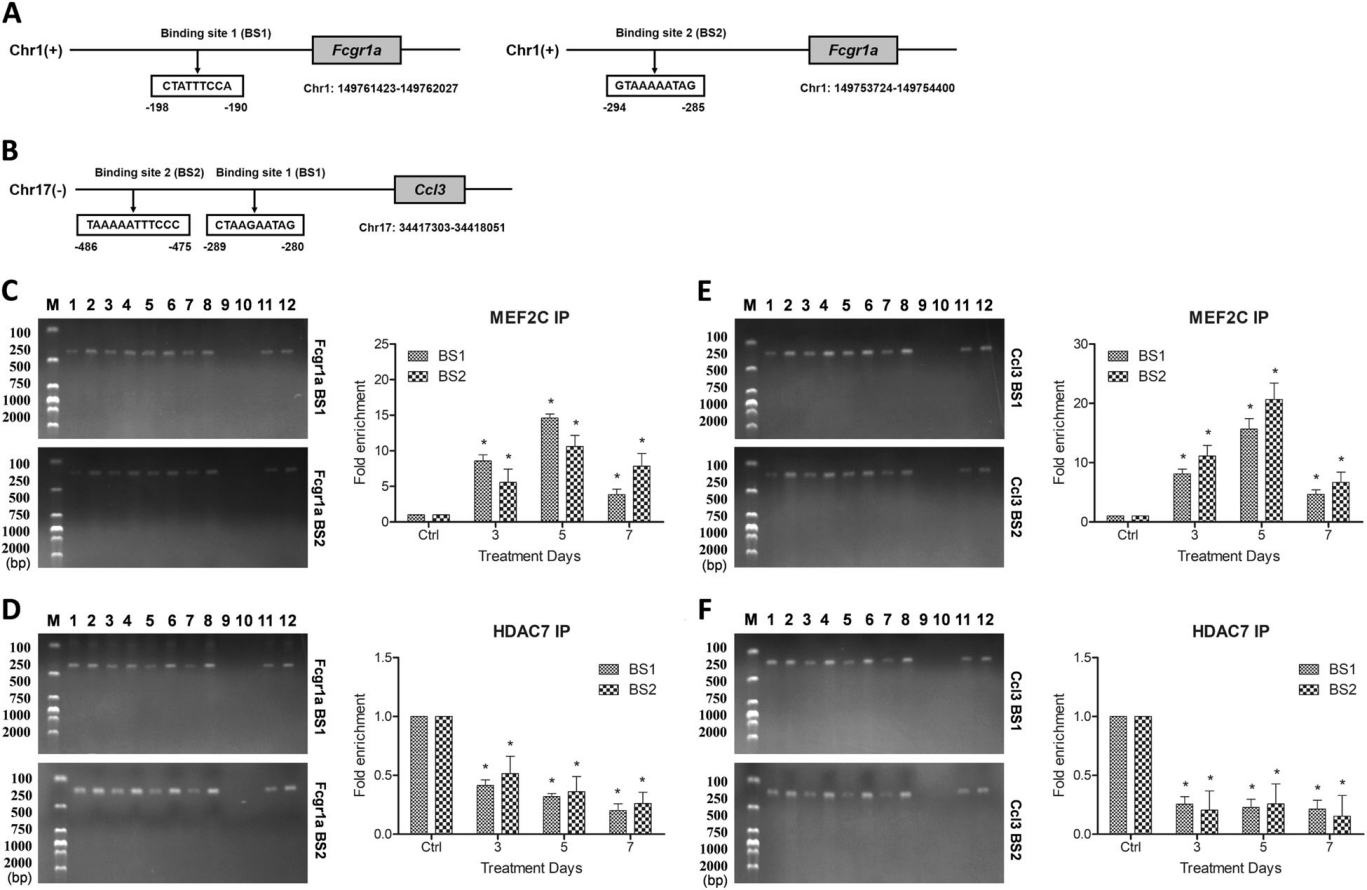
CC1007 inhibits HDAC7:MEF2C interaction on the promotor of monocyte–macrophage genes. **a**, **b** Putative binding sites in the *Fcgr1a* and *Ccl3* promoter of MEF2C. Chromatin immunoprecipitation (ChIP) experiments to detect MEF2C and HDAC7 binding to its consensus site in the *Fcgr1* and *Ccl3* gene promoter are shown. Nalm-6 cells were incubated with CC1007 (1.25 μM) for 7 days, and DNA binding was determined in nuclear extracts using ChIP. An anti-MEF2C antibody or anti-HDAC7 antibody was used for ChIP experiments to determine the composition of the DNA binding complex. Precipitated chromatin DNA was subjected to qPCR or end-point PCR, and fold changes of enrichment were assessed and compared with the control. **c**–**f** Chromatin immunoprecipitation experiments showing the enrichment of HDAC7 and MEF2C to putative MEF2 binding sites on the *Fcgr1a* and *Ccl3* gene loci in Nalm-6 cells, and CC1007 could decrease the enrichment of HDAC7 on the promotor of *Fcgr1a* and *Ccl3* genes accompanied with increase of MEF2C on the consensus site. The results using ChIP-end-point PCR were shown in the left panels of **c**–**f**, 1: MEF2C or HDAC7 antibody (Ctrl); 2: input (Ctrl); 3: MEF2C or HDAC7 antibody (3 days treatment); 4: input (3 days treatment); 5: MEF2C or HDAC7 antibody (5 days treatment); 6: input (5 days treatment); 7: MEF2C or HDAC7 antibody (7 days treatment); 8: input (7 days treatment); 9: negative control antibody; 10: negative control input; 11: positive control antibody; and 12: positive control input. The results using ChIP-qPCR were shown in the right panels of **c**–**f**. The results are representative of three independent experiments. Asterisks denote significant (*P* < 0.05) differences relative to controls by one-way ANOVA.

We performed chromatin immunoprecipitation (ChIP) to check whether HDAC7 is recruited to the promotors of monocyte–macrophage-specific genes on Nalm-6 cells by MEF2C. Based on Genomatix bioinformatics analysis, the promotors of the *Fcgr1a* and *Ccl3* genes contain putative MEF2C binding sites (Fig. 7a, b). We showed that both HDAC7 and MEF2C were specifically enriched at the identified putative MEF2C binding domain on *Fcgr1a* (Fig. 7c, d) and *Ccl3* (Fig. 7e, f) genes in untreated Nalm-6 cells. Importantly, CC1007 could enhance the enrichment of MEF2C at the binding site and reduce the binding of HDAC7 at the same site (Fig. 7c, f). These results suggest that HDAC7 is recruited to the promotors of monocyte–macrophage-specific genes via interactions with MEF2C, which leads to the inhibition of lineage differentiation regulatory genes. Apparently, CC1007 can “switch off” the transcription inhibition effect of HDAC7 and “release” the signals of monocyte–macrophage-specific gene expression by suppressing the binding of HDAC7 and MEF2C.

## Discussion

**Fig. 8:**
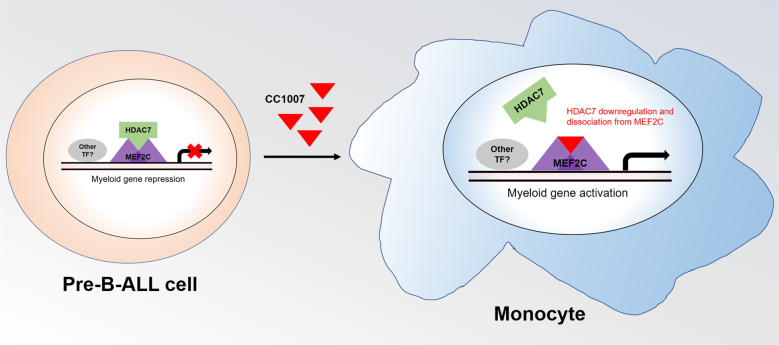
Schematic diagram for CC1007-induced BCR-ABL1^−^ pre-B-ALL cells differentiation toward monocyte. HDAC7 interacts with MEF2C in the nucleus of BCR-ABL1^−^ pre-B-ALL cells, and they are enriched at the same site on the promotor of monocyte–macrophage-specific genes. CC1007 could induce pre-B-ALL cell differentiation toward the monocyte by downregulation of HDAC7 and inhibiting HDAC7/MEF2C interaction.

In this study, we demonstrated that CC1007 had obvious antileukemic activity toward pre-B-ALL cells in vitro and induces BCR-ABL1^−^ pre-B-ALL cells cross-lineage differentiation toward monocytic lineage (Fig. 8); it also displays a strong antileukemic effect on primary pre-B-ALL xenografts and significantly prolonged median survival time of pre-B-ALL-bearing mice. In terms of the retained antileukemic effect, CC1007 had a minor effect on the activity of normal hematopoiesis.

It has been documented that an alternative therapeutic strategy for acute myeloid leukemia (AML) is differentiation inducement in promyelocytic leukemic cells, which is not solely dependent on promoting cell apoptosis. Acute promyelocytic leukemia is a typical example of the effective induction of differentiation by all-*trans* retinoic acid (ATRA)^35,36^. In ALL, a less effective differentiation-inducing agent is used for therapy. In the present study, we showed that when BCR-ABL1^−^ pre-B-ALL cells were exposed to low concentrations of CC1007 (≤1.25 μM), the cross-lineage differentiation of BCR-ABL1^−^ pre-B-ALL cells was switched on and cell apoptosis was not activated. To a certain degree, these dose-dependent dual effects of CC1007 are similar to those of ATRA or arsenic trioxide^37–40^, where antileukemic effects apparently promote a transformation that ranges from differentiation (low dose) to apoptosis (high dose) with decreased Bcl-2 expression or increased H_2_O_2_ production^38–40^. In our study, a low dose of CC1007 (≤1.25 μM) did not significantly induce BCR-ABL1^−^ pre-B-ALL cells apoptosis and did not influence the expression of apoptosis-regulating molecules, such as BCL-2, Bax, caspase-3 and -9, and cytochrome *C*, suggesting that the apoptosis pathway was not activated. Interestingly, no significant apoptosis was observed even after 7 days of low dose of CC1007 intervention, but the growth of BCR-ABL1^−^ pre-B-ALL cells was obviously inhibited, indicating that apoptosis is not involved in mechanisms of cell growth inhibition by low dose of CC1007.

Cell cycle arrest is an essential early event for differentiation. It is known that cell cycle progression, including G0/G1-phase arrest, is regulated by cyclin E1, cyclin A, or p21CIP1 molecular targets. Our results indicated that low dose of CC1007 could arrest the cell cycle at the G0/G1 phase and suppress cyclin E1, cyclin A, and c-Myc protein signaling or upregulate p21CIP1 expression in BCR-ABL1^−^ pre-B-ALL cell lines, confirming that low dose of CC1007 could promote the differentiation signal in pre-B-ALL cells. It has been reported that c-Myc plays an important role in cell cycle progression, differentiation, and apoptosis via the activation of cyclin D2 and CDK4 and inhibition of p15, p21CIP1, and p27 expression^41–44^. The mechanisms of CC1007-induced differentiation may partially be involved in c-Myc-mediated cell fate choice. p21CIP1 is not only a negative regulator of G1-phase cell cycle progression but also a player in determining cell terminal differentiation^45^, which is consistent with our finding that increased p21CIP1 expression under CC1007 treatment may contribute to pre-B-ALL cell cycle arrest and cross-lineage differentiation.

Several reports have described a potential role for HDAC7 in hematological malignancies. Rad et al.’s^28^ study showed HDAC7 is a target gene in hematopoietic cancers using a PiggyBac transposon screening in mice. HDAC7 has been shown to be overexpressed in childhood B-ALL, pediatric AML and chronic lymphocytic leukemia (CLL)^29,46,47^. However, HDAC7 has been found to be underexpressed in pro-B-ALL and B cell lymphoma^33^. Interestingly, loss of HDAC7 is not observed in most pre-B-ALL^33^. These observations are consistent with our work that showed HDAC7 is overexpressed in both BCR-ABL1^−^ and BCR-ABL1^+^ primary pre-B-ALL when compared to normal control. HDAC7 specifically interacts with MEF2C in pre-B cells and is recruited to MEF2C binding sites located at the promotors of genes related to macrophage function^26^. In addition, HDAC7 localizes to the nucleus and interacts with MEF2C in B cell malignancies^33^. As CC1007 is designed to inhibit class IIa HDACs by blocking their interaction with MEF2C, we postulate that CC1007 may interfere in the interaction between HDAC7 and MEF2 in pre-B-ALL cells. In fact, CC1007, as a BML-210 analog, can disrupt HDAC4:MEF2 co-localization in vitro and in vivo, presumably by inhibiting the HDAC4:MEF2 interaction outside the catalytic site of HDAC^30^. Our results demonstrated that CC1007 could effectively inhibit the expression of both HDAC7 and MEF2C, and reduced HDAC7 protein aggregation in the nuclei in BCR-ABL1^−^ pre-B-ALL cells. We also confirmed that HDAC7 interacted with MEF2C in BCR-ABL1^−^ pre-B-ALL cells and that CC1007 directly interacts with intracellular MEF2C, which could weaken the HDAC7:MEF2C interaction. In addition, we found that the 5′-promotors of monocyte–macrophage-specific genes *Fcgr1* and *Ccl3* contain putative MEF2C binding sites by using bioinformatics analysis. ChIP experiments indicated that both MEF2C and HDAC7 were specifically enriched at the putative MEF2C binding site on BCR-ABL1^−^ pre-B-ALL cells. When Nalm-6 cells were exposed to low dose of CC1007, HDAC7 was diminished from the binding site with MEF2C enrichment, turning on the monocyte-specific gene activation switch. This finding partially reveals the regulating mechanism of CC1007-induced cross-lineage differentiation from a lymphoid lineage toward a monocytic lineage.

Based on the findings of this study, we therefore propose that HDAC7 appears to be a potential therapeutic target in pre-B-ALL, and this small molecular compound CC1007 could inhibit tumor growth, induce cycle arrest and differentiation of BCR-ABL1^−^ pre-B-ALL cells, and to prolong the survival of BCR-ABL1^−^ pre-B-ALL-bearing mice, partially by inhibiting HDAC7 expression and HDAC7:MEF2C interaction, indicating that CC1007 may be a promising agent for the treatment of BCR-ABL1^−^ pre-B-ALL.

## Materials and methods

### Compound

CC1007 was synthesized and provided by Prof. Lin Chen (Molecular and Computational Biology, Department of Biological Sciences, University of Southern California, LA, USA). The molecular weight of CC1007 is 404.3. A stock solution (100 mM) was prepared by dissolving CC1007 in DMSO (Sigma, St. Louis, MO, USA) and stored at −20 °C.

### Cells and cell culture

The human BCR-ABL1^−^ pre-B-ALL cell line Nalm-6 (DSMZ ACC128) and MHH-CALL-2 (DSMZ ACC341) were cultured in RPMI-1640 medium with 10% fetal bovine serum at 37 °C in a humidified incubator with 5% CO_2_. MNCs in BM from pre-B-ALL patients were isolated by Ficoll centrifugation. Leukemic cells from MNCs were isolated and purified with a CD34 selection kit (Miltenyi Biotec GmbH, Germany). Primary pre-B-ALL cells were cultured in serum-free medium (StemPro®-34 SFM Complete Medium, Thermo Fisher Scientific, Cat. #10639011) in the presence of granulocyte–macrophage colony-stimulating factor (25 ng/ml), stem cell factor (100 ng/ml), and interleukin-3 (50 ng/ml). These factors were purchased from Thermo Fisher Scientific. Pre-B-ALL was diagnosed according to the World Health Organization criterion (2008). The study was approved by the Ethical Committee at the Second Xiangya Hospital, Central South University, and all patients gave written informed consent according to the Declaration of Helsinki.

### Immunofluorescence localization

BCR-ABL1^−^ pre-B-ALL cells were fixed and permeated. Staining was performed according to conventional immunohistochemistry using anti-HDAC7 and anti-MEF2C monoclonal antibodies, fluorescein isothiocyanate-conjugated immunoglobulin G (IgG), and rhodamine-conjugated IgG antibodies. DAPI (4′,6-diamidino-2-phenylindole) staining was also included in the experiment. After staining, cells were spread and enriched on glass slides by a cytospin device (StatSpin, Westwood, MA). Fluorescence signals were detected and photographed using a Leica TCS SP5 II microscope.

### Co-immunoprecipitation

Supernatants of cell lysates were incubated with anti-HDAC7 or anti-MEF2C antibody for 1 h at 4 °C, and then added 20 μl of protein G/A agarose beads (Santa Cruz Biotechnology, Dallas, TX) overnight at 4 °C. Beads were washed with cell lysis buffer and bound proteins were eluted with 2× loading sample buffer and analyzed by Western blot with indicated antibodies.

### ChIP assays

A ChIP assay was carried out according to the instructions for the Pierce^TM^ Agarose ChIP kit (Cat. #26156, Thermo Scientific). (For more details, see Supplementary Methods.)

### siRNA-mediated RNA interference

Double-stranded siRNA (RiboBio) to silence endogenous HDAC7 expression in Nalm-6 and MHH-CALL-2 cells targeted human Hdac7 mRNA (sequence: CTGCGCTATAAGCCCAAGA). Control siRNA (RiboBio) was used to control for possible non-specific effects of RNA interference. Cells were transfected with siRNA using the riboFECT™ CP (RiboBio) reagent and incubated for 72 h before continuing with the assay, and the reverse transfection method was used to reach optimal efficiency. mRNA and protein levels were examined by quantitative reverse transcription PCR (RT-qPCR) and Western blot experiments 72 h after siRNA transfection, respectively.

### Cellular thermal shift assay

CETSA and the isothermal dose–response fingerprint (ITDRF_CETSA_) were performed as described previously in Jafari et al.^48^ (For more details, see Supplementary Methods.)

### Pre-B-ALL xenograft mouse model

Antileukemic effect of CC1007 in vivo was performed in NOD/SCID immunodeficient mice engrafted with primary human pre-B-ALL cells^49^. (For more details, see Supplementary Methods.)

### Statistical analysis

All data were expressed as the mean ± SEM. Statistical analysis of multiple-group comparisons was performed by one-way analysis of variance followed by the Bonferroni post hoc test. Two-tailed *t* tests (equal variance) were used to determine statistical significance in comparisons between two groups. The survival of NOD/SCID mice was evaluated by Kaplan–Meier curves using the log-rank test to compare the differences. *P* < 0.05 was considered significant. For the CETSA experiments, data were analyzed in the GraphPad Prism software. The ITDRF_CETSA_ data were fitted using a sigmoidal (variable slope) curve fit.

## Supplementary information

Supplemental file

Supplementary figure legends

Supplementary figure 1

Supplementary figure 2

Supplementary figure 3

Supplementary table legends

Supplementary table 1

Supplementary table 2

Supplementary table 3
