# Supplementary material for: A small molecular compound CC1007 induces cross-lineage differentiation by inhibiting HDAC7 expression and HDAC7/MEF2C interaction in BCR-ABL1− pre-B-ALL
Source: Cell Death Dis. 2020 Sep 10;11(9):738. doi: 10.1038/s41419-020-02949-1 (PMC7483467; doi:10.1038/s41419-020-02949-1)
Supplement: Supplementary file 1 — Supplemental file [file 41419_2020_2949_MOESM1_ESM.docx]

**Supplementary Information**

**A small molecular compound CC1007 induces cross-lineage differentiation by inhibiting HDAC7 expression and HDAC7/MEF2C interaction in BCR-ABL1^-^ pre-B-ALL**

**Materials and Methods**

**Reagents and antibodies**

Antibodies used in the present study are as follows: Anti-Caspase-3 (#9662; 1:1000), Anti-Cleaved Caspase-3 (#9664; 1:1000), Anti-Caspase-9 (#9504; 1:1000), Anti-Cleaved Caspase-9 (#9509; 1:1000), Anti-Bcl-2 (#4223; 1:1000), Anti-Bax (#5023; 1:1000), Anti-Cytochrome C (#11940; 1:1000), Anti-Cyclin E1 (#20808; 1:1000), Anti-CDK4 (#12790; 1:1000), Anti-CDK2 (#18048; 1:1000), Anti-p21 (#2947; 1:1000), Anti-c-Myc (#18583; 1:1000) were purchased from Cell Signaling Technology (Danvers, MA). Anti-Cyclin A (#sc-271682; 1:1000), Anti-E2A (#sc-349; 1:1000) and anti-IKAROS (#sc-13039; 1:1000) mAbs were purchased from Santa Cruz Biotechnology (Santa Cruz, CA). Anti-HDAC7 (#ab166911; 1:1000), anti-p-HDAC7 (#ab111390; 1:1000), anti-HDAC7 (ChIP Grade) (#ab50212; 1:1000)and anti-MEF2C (#ab79436；1:1000) mAbs were from Abcam (Hong Kong, China). Anti-β-actin (#A5441; 1:5000) was from Sigma-Aldrich. Phycoerythrin (PE)-conjugated anti-CD34 (#348057), fluorescein isothiocyanate (FITC)-conjugated anti-CD14 (#555397), allophycocyanin (APC)-conjugated anti-CD11b (#550019), and PerCP-conjugated anti-CD45 (#652803) mAbs were from BD Bioscience (San Jose, CA). The apop­tosis detection kit, cell cycle detection kit, and nuclear and cytoplasmic protein extraction kit were purchased from KeyGEN (Nanjing, China). The mitochondria isolation kit and chromatin immunoprecipitation agents were from Thermo Scientific (Waltham, MA).

**Immunoblotting analysis**

RIPA extracts were fractionated on 10% sodium dodecyl sulfate polyacrylamide gels, electroblotted to polyvinylidene difluoride membranes and reacted with various primary antibodies. Signals were detected using ChemiDoc™ XRS+ System (Bio-Rad Laboratories, Inc, Hercules, CA).

**RT-qPCR assay**

RNA was extracted using Trizol extraction (Invitrogen) and cDNA synthesyzed using PrimeScript^®^ 1st Strand cDNA Synthesis Kit (Takara Bio Inc). RT-qPCR were performed in triplicate using SYBR^®^ Premix Ex TaqTMⅡ(Takara Bio Inc). PCR reactions were run and analyzed using the LightCycler 96 Detection System (Roche). Primers sequences were listed in supplementary table 1.

**Chromatin immunoprecipitations assays**

A chromatin immunoprecipitation (ChIP) assay was carried out according to the instructions of the Pierce^TM^ Agarose ChIP kit (Cat#26156, Thermo Scientific). We designed specific primers spanning putative MEF2C binding sites on the promotors of the Fcgr1 and Ccl3 genes. Chromatin prepared from Nalm-6 cells with 1.25 μM CC1007 treatment was subjected to ChIP with antibodies specific for MEF2C and HDAC7. CC1007-treated or untreated Nalm-6 cells were crosslinked for 10 min in 1% formaldehyde solution. Crosslinked chromatins were prepared and lysed to an average size of 500 bp by Micrococcal Nuclease. Chromatin-protein complexes were immunoprecipitated with an antibody specific to HDAC7, MEF2C or isotype-matched polyclonal IgG. Input DNA was precipitated, which was followed by quantitative RT- PCR or PCR with specific primers. The primer sequences used for amplification of the target genes are list in supplementary table 2.

**Cellular thermal shift assay (CETSA)**

For CETSA, aliquoted Nalm-6 cells and primary BCR-ABL1^-^ pre-B-ALL cells were treated for 2 h with 10 μM CC1007 or DMSO. Cells were harvested and heated for 3 min at designated temperatures (ranging from 40 to 67℃) for 3 min in a Veriti thermal cycler (Life Technologies), which was followed by cooling for 3 min at room temperature. Cell suspensions were freeze-thawed three times using liquid nitrogen. The soluble fraction (lysate) was separated from cell debris by centrifugation at 20,000×g for 20 min at 4 ℃. Soluble proteins were used for SDS-PAGE and western blot analysis. For the ITDRF_CETSA_ in cell experiments, CC1007 was serially diluted to generate a 10-point dose-response curve between each point. Nalm-6 cells and primary BCR-ABL1^-^ pre-B-ALL cells were treated with each respective compound concentration and DMSO as a control in 100-μL aliquots in 0.2-mL tubes for 2 h in an incubator at 37℃ and 5% CO2. The cell aliquots were heated at 52℃ and analyzed with western blot following the procedure described above.

**Pre-B-ALL xenograft mouse model**

Antileukemic effect of CC1007 in vivo was performed in nonobese diabetic/severe combined immunodeficiency (NOD/SCID) immunodeficient mice engrafted with primary human pre-B-ALL cells. Female NOD/SCID mice (Beijing, China) were housed in a dedicated pathogen-free environment. All animal experiments were approved by the Institutional Review Board of The Second XiangYa Hospital, Central South University. On the day of inoculation, 4-week-old mice received 250 cGy of total body irradiation at a dose rate of 100 cGy/min. Primary MNCs in BM from one patient with BCR-ABL1^-^ pre-B-ALL (Blasts in BM: 86%) were injected into each mouse via tail vein (2×10^7^ cells per mouse). When the percentage of human CD45^+^ leukemic cells in the peripheral blood increased to 1%, the mice were categorized randomly into four groups (7 mice per group). The mice were fed CC1007 (100 mg/kg or 150 mg/kg) by intragastric administration every day or cyclophosphamide (CTX, as positive control; 25 mg/kg) by intraperitoneal injection twice a week with an untreated group as a negative control. The general conditions of the mice and weight changes were observed and recorded, and the percentage of human CD45^+^ leukemic cells in peripheral blood was detected using flow cytometer each week. The survival time of mice was monitored from the first day of drug administration until death.
