# Supplementary material for: A small molecular compound CC1007 induces cross-lineage differentiation by inhibiting HDAC7 expression and HDAC7/MEF2C interaction in BCR-ABL1− pre-B-ALL
Source: Cell Death Dis. 2020 Sep 10;11(9):738. doi: 10.1038/s41419-020-02949-1 (PMC7483467; doi:10.1038/s41419-020-02949-1)
Supplement: Supplementary file 2 — Supplementary figure legends [file 41419_2020_2949_MOESM2_ESM.docx]

**Supplementary Figure 1.**

Expression of HDAC7 in MNCs from 3 patients with BCR-ABL1^-^ pre-B-ALL. Red square indicates the sample from one patient with BCR-ABL1^-^ pre-B-ALL (Pre-B-ALL#1) for mouse model. Asterisks denote statistically significant (*P*<0.05) differences compared with controls by 2-tailed Student t-tests.

**Supplementary Figure 2.**

Effect of CC1007 on the proliferation of human primary BCR-ABL1^-^ pre-B-ALL cells in a xenograft model. Percentage of human CD45^+^ leukemic cells in peripheral blood after two weeks of treatment with CC1007 in BCR-ABL1^-^ pre-B-ALL-bearing mice was detected using a FACS Calibur flow cytometer. Columns represent the average percent of human CD45^+^ cells from 3 independent experiments, which are shown as the mean ± SEM. Asterisks indicate statistically significant (*P*<0.05) differences compared with controls by 2-tailed Student *t*-tests. Representative images are shown in the upper panel. R1 represents white blood cells in the peripheral blood of mice. R2 represents human CD45^+^ leukemic cells. Ctrl, control; CC100, CC1007 100 mg/kg; CC150, CC1007 150 mg/kg; CTX, cyclophosphamide.

**Supplementary Figure 3.**

CC1007 induces BCR-ABL1^-^ pre-B-ALL cells apoptosis. BCR-ABL1^-^ pre-B-ALL cell lines (Nalm-6 and MHH-CALL-2) and primary CD34^+^ BCR-ABL1^-^ pre-B-ALL cells (n=3) were treated with increasing concentrations of CC1007 for 48 h, which was followed by analysis of apoptosis by staining with PI and Annexin V FITC. Annexin V-positive cells were measured by flow cytometry. Representative images are shown. The corresponding quantifications as a bar graph are showed in Figure 2A.
