# Supplementary figures and images for: A small molecular compound CC1007 induces cross-lineage differentiation by inhibiting HDAC7 expression and HDAC7/MEF2C interaction in BCR-ABL1− pre-B-ALL
Source: Cell Death Dis. 2020 Sep 10;11(9):738. doi: 10.1038/s41419-020-02949-1 (PMC7483467; doi:10.1038/s41419-020-02949-1)

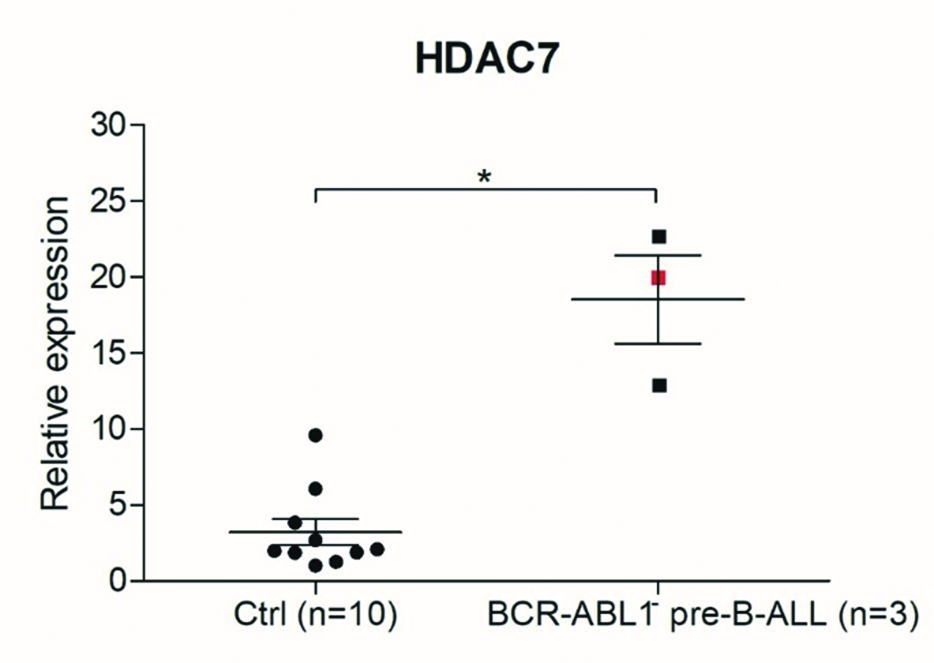

Supplement: Supplementary file 3 — Supplementary figure 1 [file 41419_2020_2949_MOESM3_ESM.tif]

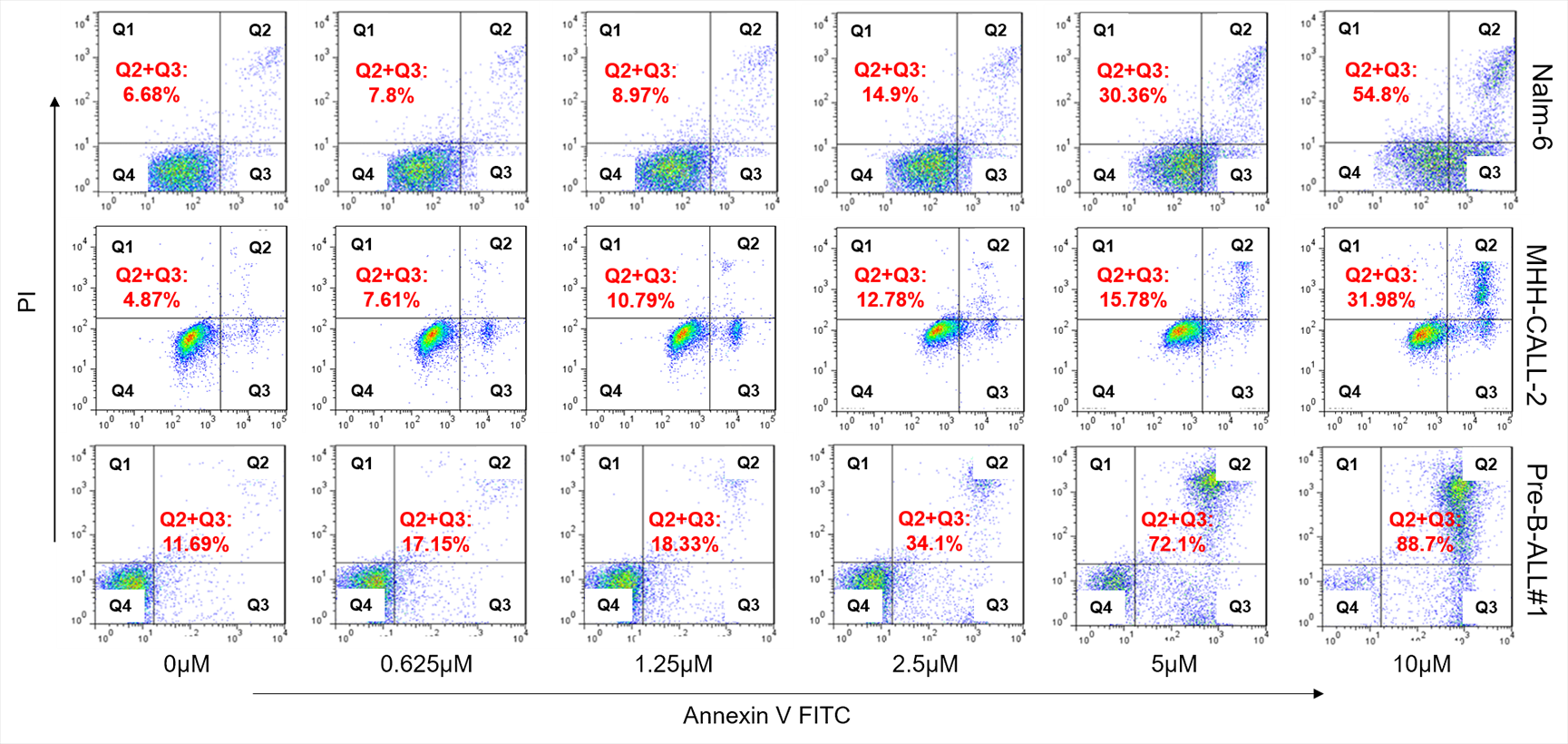

Supplement: Supplementary file 5 — Supplementary figure 3 [file 41419_2020_2949_MOESM5_ESM.tif]
