# Supplementary material for: A small molecular compound CC1007 induces cross-lineage differentiation by inhibiting HDAC7 expression and HDAC7/MEF2C interaction in BCR-ABL1− pre-B-ALL
Source: Cell Death Dis. 2020 Sep 10;11(9):738. doi: 10.1038/s41419-020-02949-1 (PMC7483467; doi:10.1038/s41419-020-02949-1)
Supplement: Supplementary file 6 — Supplementary table legends [file 41419_2020_2949_MOESM6_ESM.docx]

**Supplementary Table 1.** Sequences of oligonucleotide primers used for RT-qPCR

CDK4, cyclin-dependent kinase 4; CDK2, cyclin-dependent kinase 2; HDAC7, histone deacetylase 7; Fcgr1, Fc fragment of IgG receptor 1; Itgam, integrin subunit alpha M; Ccl3, C-C motif chemokine ligand 3; Cxcl10, C-X-C motif chemokine ligand 10; fwd, forward; rev, reverse.

**Supplementary Table 2.** Sequences of oligonucleotide primers used for qPCR or end-point PCR in ChIP

Fcgr1, Fc fragment of IgG receptor 1; Ccl3, C-C motif chemokine ligand 3; BS1, binding site 1; BS2, binding site 2; fwd, forward; rev, reverse.

**Supplementary Table 3.** Patients general information

Bone marrow aspirates were obtained from pre-B-ALL patients; BM: bone marrow; M: male, F: female; BCR-ABL1, Breakpoint cluster region (BCR)-Abelson 1 (ABL1) fusion gene.
