# Supplementary material for: A small molecular compound CC1007 induces cross-lineage differentiation by inhibiting HDAC7 expression and HDAC7/MEF2C interaction in BCR-ABL1− pre-B-ALL
Source: Cell Death Dis. 2020 Sep 10;11(9):738. doi: 10.1038/s41419-020-02949-1 (PMC7483467; doi:10.1038/s41419-020-02949-1)
Supplement: Supplementary file 7 — Supplementary table 1 [file 41419_2020_2949_MOESM7_ESM.docx]

**Supplementary Table 1.** Sequences of oligonucleotide primers used for RT-qPCR

| Gene | Sequence |
| --- | --- |
| Cyclin E1 | fwd: 5΄-TGCTGGACAAAGCCCGAGCAAAGA-3΄ |
|  | rev: 5΄-CGCTGCTCTGCTTCTTACCGCTCT-3΄ |
| Cyclin A | fwd: 5΄-TCCAAGAGGACCAGGAGAATATCA-3΄ |
|  | rev: 5΄-GTGCAACCCGTCTCGTCTTC-3΄ |
| CDK4 | fwd: 5΄-GTGTCGGTGCCTATGGGACAGTGT-3΄ |
|  | rev: 5΄-AGCCTCCAGTCGCCTCAGTAAAGC-3΄ |
| CDK2 | fwd: 5΄-CCAGAAACAAGTTGACGGGAGAGG-3΄ |
|  | rev: 5΄-CCAGTGAGAGCAGAGGCATCCA-3΄ |
| P21 | fwd: 5΄-TGTCACCGAGACACCACTGGAG-3΄ |
|  | rev: 5΄-AGCGAGGCACAAGGGTACAAGA-3΄ |
| HDAC7 | fwd: 5΄-AGAAACCCAACCTCAATGCCATCC-3΄ |
|  | rev: 5΄-TGCGGTCACTGCCTCCACTT-3΄ |
| Fcgr1 | fwd: 5΄-GCATCGCTACACATCAGCAGGAAT-3΄ |
|  | rev: 5΄-GCAGCCTCGCACCAGTATAACC-3΄ |
| Itgam | fwd: 5΄-GTCCCAGACGGAGACCAAAGTG-3΄ |
|  | rev: 5΄-TGTCCTTGTATTGCCGCTTGAAGA-3΄ |
| Ccl3 | fwd: 5΄-CACATTCCGTCACCTGCTCAGA-3΄ |
|  | rev: 5΄-GGCTGCTCGTCTCAAAGTAGTCA-3΄ |
| Cxcl10 | fwd: 5΄-GCCTCTCCCATCACTTCCCTACAT-3΄ |
|  | rev: 5΄-TGAAGCAGGGTCAGAACATCCACT-3΄ |
| β-actin | fwd: 5΄-TTCCAGCCTTCCTTCCTGGG-3΄ |
|  | rev: 5΄-TTGCGCTCAGGAGGAGCAAT-3΄ |

CDK4, cyclin-dependent kinase 4; CDK2, cyclin-dependent kinase 2; HDAC7, histone deacetylase 7; Fcgr1, Fc fragment of IgG receptor 1; Itgam, integrin subunit alpha M; Ccl3, C-C motif chemokine ligand 3; Cxcl10, C-X-C motif chemokine ligand 10; fwd, forward; rev, reverse.
