# Supplementary material for: A small molecular compound CC1007 induces cross-lineage differentiation by inhibiting HDAC7 expression and HDAC7/MEF2C interaction in BCR-ABL1− pre-B-ALL
Source: Cell Death Dis. 2020 Sep 10;11(9):738. doi: 10.1038/s41419-020-02949-1 (PMC7483467; doi:10.1038/s41419-020-02949-1)
Supplement: Supplementary file 8 — Supplementary table 2 [file 41419_2020_2949_MOESM8_ESM.docx]

**Supplementary Table 2.** Sequences of oligonucleotide primers used for qPCR or end-point PCR in ChIP

| Gene | Sequence |
| --- | --- |
| Fcgr1a (BS1) | fwd: 5΄-AAAGGACCTGGATGCTAAACAG-3΄ |
|  | rev: 5΄-TGCCCATGTAGAAGGAGAAGTA-3΄ |
| Fcgr1a (BS2) | fwd: 5΄-GCATTAGCCAGGCATTGAAC-3΄ |
|  | rev: 5΄-TCAGGTTATGAACTCTCAGAATCC-3΄ |
| Ccl3 (BS1) | fwd: 5΄-GCTGAGGAAGCAGAATTG-3΄ |
|  | rev: 5΄-TCCTGTGAGTGTGAAGAG-3΄ |
| Ccl3 (BS2) | fwd: 5΄-GGACCCTGAGTTGTGCAA-3΄ |
|  | rev: 5΄-TGCCCGTGTCCTTCTGAA-3΄ |

Fcgr1, Fc fragment of IgG receptor 1; Ccl3, C-C motif chemokine ligand 3; BS1, binding site 1; BS2, binding site 2; fwd, forward; rev, reverse.
