# Supplementary material for: A small molecular compound CC1007 induces cross-lineage differentiation by inhibiting HDAC7 expression and HDAC7/MEF2C interaction in BCR-ABL1− pre-B-ALL
Source: Cell Death Dis. 2020 Sep 10;11(9):738. doi: 10.1038/s41419-020-02949-1 (PMC7483467; doi:10.1038/s41419-020-02949-1)
Supplement: Supplementary file 9 — Supplementary table 3 [file 41419_2020_2949_MOESM9_ESM.docx]

**Supplementary Table 3.** Patients general information

| **Sample** | **Type** | **Age(Year)/Sex** | **Clinical status** | **Blasts(%)** | **Differentiation antigen expression** | **BCR-ABL1** |
| --- | --- | --- | --- | --- | --- | --- |
| 1 | BM | 45/F | Diagnosis | 85.00 | CD34, CD10, CD19, HLA-DR, CD13 | - |
| 2 | BM | 42/F | Diagnosis | 69.00 | CD34, CD10, CD19, HLA-DR | - |
| 3 | BM | 15/M | Diagnosis | 87.00 | CD34, CD10, CD19, HLA-DR | + |
| 4 | BM | 22/M | Diagnosis | 79.00 | CD34, CD10, CD19, HLA-DR | + |
| 5 | BM | 37/M | Diagnosis | 61.00 | CD34, CD10, CD19, HLA-DR, CD13 | + |
| 6 | BM | 23/M | Diagnosis | 74.00 | CD34, CD10, CD19, HLA-DR | - |
| 7 | BM | 31/M | Diagnosis | 86.00 | CD34, CD10, CD19, HLA-DR, CyCD79a | - |
| 8 | BM | 15/F | Diagnosis | 86.50 | CD34, CD10, CD19, HLA-DR, CD13, CD7 | + |
| 9 | BM | 53/F | Diagnosis | 82.50 | CD34, CD10, CD19, HLA-DR | - |
| 10 | BM | 26/F | Diagnosis | 82.50 | CD34, CD10, CD19, HLA-DR | - |
| 11 | BM | 19/F | Diagnosis | 87.50 | CD34, CD10, CD19, HLA-DR, CD13 | - |
| 12 | BM | 24/M | Diagnosis | 78.50 | CD34, CD10, CD19, HLA-DR, CD13 | + |
| 13 | BM | 15/M | Relapsed | 80.00 | CD34, CD10, CD19, HLA-DR, CD13 | + |
| 14 | BM | 41/F | Diagnosis | 67.70 | CD34, CD10, CD19, HLA-DR, CD13 | - |
| 15 | BM | 16/F | Diagnosis | 76.00 | CD34, CD10, CD19, CD14, CD3, CD33, CD7 | + |
| 16 | BM | 30/M | Diagnosis | 92.50 | CD34, CD10, CD19, HLA-DR, CD13 | + |
| 17 | BM | 47/M | Diagnosis | 86.00 | CD34, CD10, CD19, HLA-DR, cTDT, CyCD79a | + |
| 18 | BM | 47/F | Diagnosis | 84.50 | CD34, CD10, CD19, HLA-DR | - |
| 19 | BM | 16/F | Diagnosis | 81.00 | CD34, CD10, CD19, HLA-DR, CD13 | - |
| 20 | BM | 41/F | Diagnosis | 91.00 | CD34, CD10, CD19, HLA-DR, CD13 | - |
| 21 | BM | 18/M | Diagnosis | 73.50 | CD34, CD10, CD19, HLA-DR, CD13, CyCD79a | + |
| 22 | BM | 33/M | Diagnosis | 70.00 | CD34, CD10, CD19, HLA-DR, CD33, CD20 | - |
| 23 | BM | 21/M | Diagnosis | 77.00 | CD34, CD10, CD19, HLA-DR, CyCD79a | - |
| 24 | BM | 48/M | Diagnosis | 61.00 | CD34, CD10, CD19, HLA-DR, CD33, CD13 | - |
| 25 | BM | 25/M | Diagnosis | 50.00 | CD34, CD10, CD19, HLA-DR, CD22, CD15 | - |
| 26 | BM | 24/M | Diagnosis | 75.50 | CD34, CD10, CD19, HLA-DR, CD20, CD13 | - |
| 27 | BM | 15/M | Diagnosis | 86.00 | CD34, CD10, CD19, HLA-DR, CD20 | + |
| 28 | BM | 21/M | Diagnosis | 88.50 | CD34, CD10, CD19, HLA-DR, CD13 | - |
| 29 | BM | 62/M | Diagnosis | 88.00 | CD34, CD10, CD19, HLA-DR, CD13 | + |
| 30 | BM | 72/F | Diagnosis | 87.50 | CD34, CD10, CD19 | - |
| 31 | BM | 29/M | Diagnosis | 54.00 | CD34, CD10, CD19, HLA-DR, CD22, CD13 | + |
| 32 | BM | 33/F | Refractory | 35.00 | CD34, CD10, CD19, HLA-DR, CD33, CD13 | + |
| 33 | BM | 30/M | Diagnosis | 88.00 | CD34, CD10, CD19, HLA-DR, CD13 | + |
| 34 | BM | 39/F | Refractory | 73.00 | CD34, CD10, CD19, HLA-DR, CD13 | + |
| 35 | BM | 14/F | Diagnosis | 73.00 | CD34, CD10, CD19, HLA-DR | - |
| 36 | BM | 22/F | Diagnosis | 86.00 | CD34, CD10, CD19, HLA-DR, CD13 | + |
| 37 | BM | 31/M | Relapsed | 60.50 | CD34, CD10, CD19, HLA-DR | + |
| 38 | BM | 19/F | Diagnosis | 75.00 | CD34, CD10, CD19, HLA-DR, CD13 | - |
| 39 | BM | 18/M | Diagnosis | 94.00 | CD34, CD10, CD19, HLA-DR, CD20, CD13 | - |
| 40 | BM | 33/F | Relapsed | 93.00 | CD34, CD10, CD19, HLA-DR, CD33, CD13 | + |
| 41 | BM | 14/F | Diagnosis | 88.00 | CD34, CD10, CD19, HLA-DR, CD13, CyCD79a | - |
| 42 | BM | 66/F | Diagnosis | 87.00 | CD34, CD10, CD19, HLA-DR, CD13 | - |
| 43 | BM | 24/F | Diagnosis | 87.50 | CD34, CD10, CD19, HLA-DR, CD13 | - |
| 44 | BM | 60/M | Diagnosis | 54.00 | CD34, CD10, CD19, HLA-DR | - |
| 45 | BM | 29/M | Refractory | 68.50 | CD34, CD10, CD19, HLA-DR, CD22, CD13 | + |
| 46 | BM | 16/F | Diagnosis | 88.00 | CD34, CD10, CD19, HLA-DR, CD13 | - |
| 47 | BM | 16/M | Relapsed | 89.00 | CD34, CD10, CD19, HLA-DR, CD33, CD13 | + |

Bone marrow aspirates were obtained from pre-B-ALL patients; BM: bone marrow; M: male, F: female; BCR-ABL1, Breakpoint cluster region (BCR)-Abelson 1 (ABL1) fusion gene.
